# Kaixinsan, a Well-Known Chinese Herbal Prescription, for Alzheimer's Disease and Depression: A Preclinical Systematic Review

**DOI:** 10.3389/fnins.2019.01421

**Published:** 2020-01-14

**Authors:** Huan Fu, Zhen Xu, Xi-le Zhang, Guo-qing Zheng

**Affiliations:** Department of Neurology, The Second Affiliated Hospital and Yuying Children's Hospital of Wenzhou Medical University, Wenzhou, China

**Keywords:** Kaixinsan, Alzheimer's disease, behavioral and psychological symptoms of dementia, depression, systematic review, meta-analysis

## Abstract

Alzheimer's disease (AD), the most common cause of dementia, is highly prevalent worldwide with no modifying therapy. Behavioral and psychological symptoms of dementia (BPSD) occur in most patients with AD, and depression is one of the most common AD-related BPSD. Kaixinsan (KXS) is an ancient Chinese herbal prescription widely used to treat dementia and forgetfulness. In this systematic review, we conducted a meta-analysis to assess preclinical evidence for the effects of KXS on cognitive impairment and depression. Thirty-eight articles involving 1,050 animals were included after searching from six databases from the inception up to June 2019. The primary outcome measures were behavioral outcome. Indicators of cognitive function in AD included escape latency, time spent on the target quadrant, and the number of target platform crossings in the Morris water maze (MWM) test. Indicators of depression included number of rearing events and total distance in the open-field test, duration of immobility in the forced swim test, and sucrose consumption or sucrose preference index in the sucrose preference test. The secondary outcomes were mechanisms of KXS for treatment of AD and depression. The results showed that KXS significantly reduced escape latency (*P* < 0.01), increased time spent in the target quadrant (*P* < 0.01), and increased the number of target platform crossings (*P* < 0.01) in the MWM test in AD models compared with control. The possible mechanisms for KXS-mediated improvements in cognitive function were antioxidant activity, anti-inflammatory activity, antiapoptotic activity, neuroprotection, and synapse protection. In addition, the results demonstrated that KXS significantly increased the number of rearing instances (*P* < 0.01) in the open-field test, decreased the duration of immobility (*P* < 0.01) in forced swim test, and increased sucrose consumption or sucrose preference index (*P* < 0.01) in the sucrose preference test in depression models compared with control. The mechanisms of KXS-mediated anti-depressive effects were HPA axis regulation, antioxidant activity, anti-inflammatory activity, synapse protection, and neuroprotection. The results of this study suggested that KXS can be used to effectively treat AD and depression through multiple mechanisms, extrapolating the therapeutic potential of KXS for treating AD-related BPSD.

## Introduction

Alzheimer's disease (AD), a common progressive neurodegenerative disease with gradual onset (Karlawish et al., 2017), is the leading cause of dementia (Alzheimer's, 2016). There are currently 44 million patients with dementia worldwide, 50–75% of whom have AD (Lane et al., 2018). Approximately 5–7 million individuals are diagnosed with AD annually (Robinson et al., 2018). The cost of AD treatment and care has resulted in a considerable economic burden to families and society (Alzheimer's, 2016). Acetylcholinesterase inhibitors (Birks and Grimley Evans, 2015) and memantine (Porsteinsson et al., 2008) are used to provide symptom relief. However, disease-modifying treatments have not been developed (Lane et al., 2018).

It was estimated that ~90% of patients with AD exhibit obvious behavioral and psychological symptoms of dementia (BPSD) (Chakraborty et al., 2019), a series of behaviors and neuropsychiatric symptoms such as depression, agitation, mood disorders, sleep disturbances, psychosis, apathy, aberrant motor activity, dysphoria, delusions, and hallucinations in patients with dementia (Dyer et al., 2018). What adds insults to injury, BPSD further seriously affect survival quality of AD patients, leading to huge social burden (Moore et al., 2001).

Among mass of clinical presentations of BPSD, depression is a major symptom that occurs in 54–64% of patients with dementia (Preuss et al., 2016). Because development of BPSD can be multifactorial, a single treatment does not exist for this constellation of symptoms (Preuss et al., 2016). Current major treatments for BPSD can be divided into non-pharmacological approaches such as music therapy, touch therapies or massage, and pharmacological approaches such as cognitive enhancers, antipsychotics, mood stabilizers, and antidepressants (Gitlin et al., 2001). However, non-pharmacological approaches are rarely used because of lack of provider training, professional staff, or equipment (Cohen-Mansfield et al., 2013). Pharmacological treatments are often associated with side effects and other health risks (Preuss et al., 2016). Thus, it is necessary to find a comprehensive treatment for both AD and BPSD.

Traditional Chinese medicine (TCM) formulae is a combination of various kinds of herbs, could express synergistic efficacies through multiple targets. For thousands of years, TCM has been playing an indispensable role in disease treatment (Zhang et al., 2013). Kaixinsan, a traditional Chinese herbal prescription, was first used to treat dementia and forgetfulness in *Prescriptions Worth a Thousand Pieces of Gold* (*BeijiQianjinYaofang*), written by Sun Si-Miao in the Tang dynasty (618–907 A.D.). Kaixinsan is comprised of four herbs, Ginseng Radix (*Panax ginseng* C. A. Mey.), Polygalae Radix (*Polygala tenuifolia* Wild.), Poria [*Poriacocos* (Schw.) Wolf], and Acori Tatarinowii Rhizoma (*Acorustatarinowii* Schott), in a 4:4:2:1 ratio. Previous clinical trials showed that KXS ameliorated clinical symptoms of patients with dementia (Liu Y. T. et al., 2015) and depression (Bao et al., 2011). Pharmacological studies indicated that KXS significantly improved cognitive function (Chu et al., 2016b) and reduced depressive-like behavior (Dou, 2017).

KXS is a traditional prescription used to treat dementia and forgetfulness for thousands of years in east Asia. However, the clinical trials of KXS specifically used in BPSD are still insufficient. Preclinical studies could illustrate possible mechanisms and provide evidence for clinical application. Although there are numerous preclinical experiments, there is no systematic review of KXS for AD or depression at present. A systematic review of preclinical studies is an ethical approach to synthesize preclinical evidence, may identify confounding factors across animal studies (Ritskes-Hoitinga et al., 2014). Thus, the present study was conducted focusing on animal experiments, with the goal of confirming that KXS might be effective to BPSD.

## Methods

### Database and Literature Search Strategy

The following six databases were searched: Web of Science, PubMed, the Cochrane Library, Wanfang database, Chinese National Knowledge Infrastructure (CNKI), and VIP Journals Database from inception to June 2019. Studies reporting the use of KXS to treat cognitive impairment or depression in animals were identified. The search terms were as follows: 1. kaixin*; 2. kai xin; 3. OR/1-2.

### Study Selection

Two investigators screened the titles and/or abstracts independently. The inclusion criteria were as follows: (1) animal studies that assessed the effectiveness of KXS for treatment of cognitive impairment and depression; (2) experimental group received KXS as a monotherapy at any dose; (3) comparator interventions were non-functional liquids (normal saline or distilled water) or positive drugs; (4) no restriction on animal species, sex, age, or weight. Exclusion criteria were as follows: (1) clinical articles, case reports, reviews, comments, abstracts, and *in vitro* studies; (2) *in vitro* models; (3) cognitive impairment induced by vascular dementia, Parkinson's disease, or alcohol. In the case of duplicate publications from one study, we chose the articles with the earliest publication dates or with the largest sample sizes.

### Data Extraction

The following details were extracted by two independent investigators per our previous systematic review (Ma et al., 2018): (1) first author name and publication year; (2) animal information for each study including species, sex, number, and weight; (3) modeling approach of animal models and anesthetic used in the model; (4) characteristics of intervention, including timing of initial treatment, duration of treatment, method and dosage of treatment, and corresponding control group information; (5) outcome measures and corresponding p-values. For each comparison, the mean value and standard deviation were extracted from each treatment and control group in every study. In the case of studies where the data were only expressed graphically, we attempted to contact the authors for detailed data, or we calculated the data ourselves using Engauge Digitizer 10.11 software. The result of the highest dose was included when the treatment group received different doses of the target drug. The data from the middle time point was selected when data were collected at multiple time points.

### Quality Assessment

Assessment of methodological quality of the included articles was conducted by two independent investigators according to our previous study (Ma et al., 2018) with one minor change: aging models were considered appropriate. Every item was assigned one point, and the sum was used as the quality score. Divergences were addressed through discussion or consultation with the corresponding investigator.

### Statistical Analysis

Data analysis was conducted using RevMan 5.3 software. All outcome measures were entered as continuous data. The combined overall effect sizes were estimated using standard mean differences (SMD). *I*^2^ values were used to determine whether the studies used fixed effects models (*I*^2^ < 50%) or random effects models (*I*^2^ > 50%). The efficacy of KXS and its bioactive ingredients was estimated using SMD and 95% confidence intervals (CI). Publication bias was assessed using funnel plots. Subgroup analysis was used to identify potential confounding factors that may have resulted in heterogeneity of outcome measures. Sensitivity analysis was conducted by excluding one study at a time from all studies to confirm that the results were stable. Heterogeneity among individual studies was assessed using the I^2^ statistic. Statistical significance was indicated by *P* < 0.05.

## Results

### Study Inclusion

**Figure 1:**
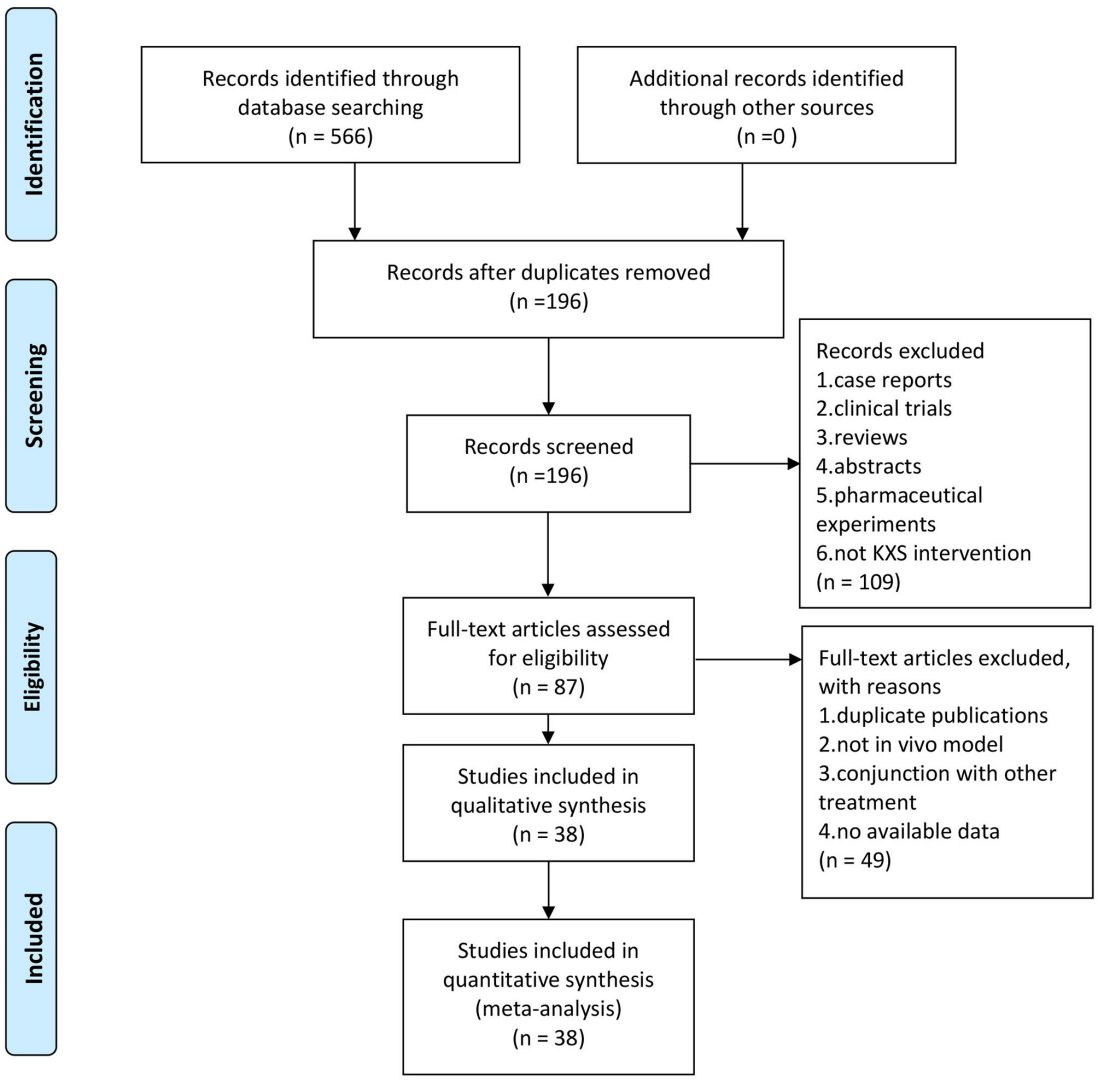
The PRIMSA flow diagram of study selection.

Our search produced 566 hits across six databases. One hundred ninety-six articles remained after excluding irrelevant articles and duplicates. One hundred nine manuscripts were removed after scanning the titles and abstracts because they were clinical articles, case reports, comments, reviews, or pharmaceutical experiments. After reading the full text of the remaining 87 articles, 49 were excluded because they were duplicate publications, not *in vivo* model, or KXS was administered in conjunction with other treatments. Finally, 38 eligible studies which involved 1,050 animals were selected (Figure 1), in which 17 studies used depression models and 21 studies used cognitive impairment models.

### Characteristics of Included Studies

The characteristics of the 38 included studies are summarized in Table 1. The included articles were published between 1999 and 2019. Thirteen (34.2%) articles were published in English and 25 (65.8%) were published in Chinese. Twenty-one (55.3%) studies used rat models, of which 12 used Wistar rats and nine used Sprague-Dawley (SD) rats. The remaining 17 (44.7%) studies used mouse models, including ICR (*n* = 6), APP/PS1 (*n* = 1), C57BL/6J (*n* = 1), SAMP8 (*n* = 3), and Kunming mice (*n* = 8). Seventeen (44.7%) studies induced cognitive impairment using Alzheimer's disease (AD) models, and 5 (13.2%) induced cognitive impairment using an aging model. Seventeen (44.7%) studies induced depression using a chronic stress model. For anesthesia, 7 (18.4%) studies used pentobarbital, 2 (5.3%) studies used chloral hydrate, 17 (44.7%) studies did not use anesthesia, and 13 (34.2%) studies did not mention if anesthesia was used. For outcome measures, 9 (23.7%) studies evaluated escape latency in the MWM. Eight (21.1%) studies reported the number of target platform crossings in the MWM. Five (13.2%) studies reported time spent on the target quadrant in the MWM. In the open-field test, 9 (23.7%) studies presented the number of rearing events and 3 (7.9%) studies reported total distance. Thirteen (34.2%) studies evaluated sucrose consumption or sucrose preference index in the sucrose preference test. Six (15.8%) studies reported duration of immobility in the forced swim test. Superoxide dismutase (SOD) was evaluated in 7 (18.4%) studies, malondialdehyde (MDA) was evaluated in 5 (13.2%) studies, acetylcholine (Ach) was evaluated in 4 (10.5%) studies, acetylcholinesterase (AChE) was evaluated in 7 (18.4%) studies, norepinephrine (NE) was evaluated in 9 (23.7%) studies, dopamine (DA) was evaluated in 9 (23.7%) studies, and 5-hydroxytryptamine (5-HT) was evaluated in 10 (26.3%) studies.

**Table 1:** Characteristics of the 38 included studies.

**References**	**Species (Sex; *n* = experimental /control group)**	**Weight**	**Modeling approach**	**Anesthetic**	**Interventionaannggeell trial group**	**Control group**	**Outcome measure**	**Intergroupaannggeell differences**
Bian et al. (2000)	SD rats (male and female,9/8)	180–230 g	Cognitive impairment induced by i.p. SCOP (5 mg/kg)	NR	KXS,i.g. 0.1, 0.6 g/kg/day for 7 days before the model	Distilled water	1. The number of errors in Y maze 2. 5-HT,5-HIAA, NE, DA 3. SOD, MDA	1. *P* < 0.01 2. *P* < 0.01 4. *P* < 0.01
Shang (2003)	Kunming mice (male and female,10/10)	16–20 g	Cognitive impairment induced by SCOP 2 mg/kg	NR	KXS,i.g. 118.5/237 mg/kg/day for 12 days before the model	Distilled water	1. Time of correct in SDT	1. *P* < 0.01
	ICR mice (male and female,12/12)	16–20 g	Cognitive impairment induced by SCOP 2 mg/kg	NR	KXS,i.g. 118.5/237 mg/kg/day for 17 days accompanying the model	Distilled water	1. The number of errors in MWM 2. Escape latency in water maze	1. *P* > 0.05 2. *P* < 0.05
	ICR mice (male and female,12/12)	16–20 g	Cognitive impairment induced by SCOP 2 mg/kg	NR	KXS,i.g. 118.5/237/355.5 mg/kg/day for 23 days before the model	Distilled water	1. AchE 2. SOD	1. *P* < 0.01 2. *P* < 0.05
	Kunming mice (male and female,12/12)	16–20 g	Cognitive impairment induced by SCOP 2mg/kg	NR	KXS,i.g. 118.5/237/355.5 mg/kg/day for 23 days before the model	Distilled water	1. ChAT	1. *P* < 0.05
Bian et al. (1999)	ICR mice (male,9/8)	18–22 g	Cognitive impairment induced by SCOP 5 mg/kg	NR	KXS,i.g. 0.1/0.3 g/kg/day for 7 days before the model	Distilled water	1. number of correct in Y maze	1. *P* < 0.01
	SD rats (male,10/10)	480–620 g	Aging model	NR	KXS,i.g. 0.1/0.3 g/kg/day for 7 days before the model	Distilled water	1. Number of correct in Y maze	1. *P* < 0.01
	ICR mice (male,9/8)	18–22 g	AD model induced by AlCl3 4 mg	NR	KXS,i.g. 0.1/0.3 g/kg/day for 3 months accompanying the model	Distilled water	1. Number of correct in Y maze 2. Number of correct in water maze	1. *P* < 0.01 2. *P* < 0.05
Zhou et al. (2008)	Kunming mice (male,12/12)	18–22 g	Cognitive impairment model induced by D-gal 150 mg/kg	NR	KXS,i.g. 0.1/0.3/0.9 g/kg/day for 6 weeks	Normal saline	1. Escape latency in MWM 2. The number of error in MWM 3. AGEs 4. SOD, MDA	1. *P* < 0.01 2. *P* < 0.01 3. *P* < 0.01 4. *P* < 0.01
Gao et al. (2010)	Kunming mice (male,15/15)	28–30 g	AD model induced by D-gal+SCOP	NR	KXS,i.g. 5, 10 g/kg/day for 12 weeks accompanying the model	Normal saline	1. Escape latency in MWM 2. Time spent in target quadrant 3. Percentage of finding the security desk 4. Escape latency in SDT 5. Number of errors in SDT 6.AchE,SOD,MDA	1. *P* < 0.05 2. *P* < 0.05 3. *P* < 0.01 4. *P* < 0.05 5. *P* < 0.01 6. *P* < 0.01
Li et al. (2016)	Kunming mice (male and female,10/10)	18–22 g	AD model induced by D-gal+sodium nitrosum	NR	KXSE, i.g. 0.892/1.785/3.570 g/kg/day for 35 days after the model	Distilled water	1. Eacape latency in MWM 2. The number of target platform crossings 3. swimming distance in target quadrant 4. Ach 5. AchE 6. Tau 7. p-Tau 8. Aβ 9.NT-proBNP	1. *P* < 0.05 2. *P* < 0.05 3. *P* < 0.05 4. *P* < 0.01 5. *P* < 0.05 6. *P* < 0.01 7. *P* < 0.05 8. *P* < 0.01 9. *P* < 0.05
Xu and Jiang (2017)	Wistar rats (male and female, 12/12)	250–300 g	AD model induced by bilateral hippocampal injection Aβ1–42 with 5 ug	Sodium pentobarbital(50 mg/kg)	KXS,i.g. 1.6/2.4/3.6 g/kg/day for 28 days after the model	Distilled water	1. Escape latency in MWM 2. Time spent in target quadrant 3. The number of target platform crossing	1. *P* < 0.05 2. *P* < 0.05 3. *P* < 0.01
Zhong (2005)	Wistar rats (male and female, 12/12)	400–450 g	AD model induced by bilateral hippocampal injection Aβ25–35 with 5 ug	pentobarbital (40 mg/kg i.p.)	KXS,i.g. 0.1, 0.3 g/kg/day for 28 days after the model	Distilled water	1.Escape latency in MWM 2. AchE 3. APP 4. bax, bcl-2 5.Trib3	1. *P* < 0.05 2. *P* < 0.05 3. *P* < 0.01 4. *P* < 0.05 5. *P* < 0.001
Shi et al. (2017b)	SAMP8 mice/SAMR1 mice (male, 10/10)	24.5–34.2 g/30.2–37.6 g	Aging model induced by Gene knockout	No need	KXS,i.g. 0.195/0.78 g/kg/day for 8 weeks after the model	Normal saline	1. The number of target platform crossings 2. Time spent in target quadrant 3.The number of errors in STD 4. Escape latency in STD 5.mt-DNA	1. *P* < 0.05 2. *P* < 0.01 3. *P* < 0.05 4. *P* < 0.01 5. *P* < 0.05
Huang et al. (1999)	ICR mice (male, 13/13)	18–22 g	AD model induced by AlCl3	NR	KXS,i.g. 0.39, 0.13 g/kg/day for 3 months accompanying the model	Distilled water	1.Number of correct in Y maze 2.Number of correct in water maze	1.*P* < 0.01 2. *P* < 0.05
Huang et al. (1998)	SD rats (male, 10/10)	480–620 g	Aging model	NR	KXS,i.g. 0.1/0.3 g/kg/day for 7 days before the model	Distilled water	1. Number of correct in Y maze 2. SOD, NE,5-HT,DA	1. *P* < 0.01 2. *P* < 0.05
Dang (2008)	SD rats (male and female, 12/11)	190–250 g	Depression model induced by chronic stress	No need	KXS,i.g. 2.7, 0.9, 0.3 g/kg/day for 53 days accompanying the model	Distilled water	1. Weight 2. Average speed in open field 3. Total distance in open field 4. Number of rearing in open field	1. *P* < 0.05 2. *P* < 0.05 3. *P* < 0.05 4. *P* < 0.05
Zhang et al. (2016)	Wistar rats (male, 10/10)	240 ± 20 g	Depression model induced by chronic stress	No need	KXS,i.g. 445 mg/kg/day for 6 weeks accompanying the model	Distilled water	1. Level of emotional arousal 2. The score of horizontal movement in open field test 3. The score of vertical movement in open field test 4. time of staying in center 5. ACTH, CRH, CORT 6. GR	1. *P* < 0.05 2. *P* < 0.05 3. *P* < 0.05 4. *P* < 0.05 5. *p* > 0.05 6. *P* < 0.01
Wang et al. (2007)	Wistar rats (male, 10/10)	150–180 g	Depression model induced by chronic stress	No need	KXS,i.g. 4, 8 g/kg/day for 21 days accompanying the model	Distilled water	1. Sucrose consumption in sucrose preference test 2. The score of horizontal movement in open field test 3. The score of vertical movement in open field test	1. *P* < 0.01 2. *P* < 0.05 3. *P* < 0.01
Liu M. et al. (2012)	SD rats (male, 10/10)	180–220 g	Depression model induced by chronic stress	No need	KXS,i.g. 1,000, 500, 250, 125 mg/kg/day for 21 days accompanying the model	Distilled water	1. Sucrose preference index in sucrose preference test 2. Total distance in open field test 3. EL in MWM 4. Time spent in target quadrant 5. The number of target platform crossings 6. 5-HT, DA,NE 7. Ach, AchE	1. *P* < 0.01 2. *P* < 0.01 3. *P* < 0.01 4. *P* < 0.01 5. *P* < 0.01 6. *P* < 0.01 7. *P* < 0.05
Liu W. W. et al. (2015)	SD rats (male, 8/8)	180–220 g	Depression model induced by chronic stress	No need	KXS,i.g. 1.785 g/kg/day after the model	Distilled water	1. Weight 2. Sucrose preference index in sucrose preference test 3. The score of horizontal movement in open field test 4. The score of vertical movement in open field test 5.NE,DA,5-HT	1. *p* > 0.05 2. *p* > 0.05 3. *p* > 0.05 4. *p* > 0.05 5. *p* > 0.05
Duan et al. (2016)	ICR mice (male and female, 10/10)	20–22 g	Depression model induced by chronic stress	No need	KXS,i.g. 10 g/kg/day for 7 days after the model	Distilled water	1. Sucrose preference index in sucrose preference test 2. Duration of immobility in forced swim test 3. NGF 4. BDNF	1. *P* < 0.05 2. *P* < 0.01 3. *p* > 0.05 4. *p* > 0.05
Zhang et al. (2018)	SD rats (male, 8/8)	170–200 g	Depression model induced by chronic stress	No need	KXS,i.g. 1.785 g/kg/day for 21 days after the model	Distilled water	1. Weight 2. Sucrose preference index in sucrose preference test 3. The score of horizontal movement in open field test 4. The score of vertical movement in open field test 5. Forced swimming test 6. SOD, MDA, CAT, GSH-Px, CRP, I-6,TNF-α	1. *P* < 0.01 2. *P* < 0.01 3. *p* > 0.05 4. *P* < 0.01 5. *P* < 0.01 6. *P* < 0.01
Dou (2017)	SD rats (male, 10/10)	200 ± 20 g	Depression model induced by chronic stress	No need	KXS,i.g. 4.5 g/kg/day for 28 days accompanying the model	Distilled water	1. Weight 2. Sucrose preference index in sucrose preference test 3. Total distance in open field test 4. Forced swimming test	1. *p* > 0.05 2. *p >* 0.05 3. *p >* 0.05 4. *p <* 0.01
Xu et al. (2019)	Kunming mice (male, 12/12)	35–40 g	Cognitive impairment induced by SCOP	NR	KXS,i.g. 0.7/1.4/2.8 g/kg/day for 14 days accompanying the model	Normal saline	1. Escape latency in MWM 2. The number of target platform crossings 3. Time spent in target quadrant 4. Y maze 5. Bax/Bcl-2,Ach,AchE,ChAT 6.SOD,MDA	1. *p <* 0.01 2. *p <* 0.01 3. *p <* 0.01 4. *p <* 0.05 5. *p <* 0.05 6. *p <* 0.01
Chu et al. (2016b)	Wister rats (male, 10/10)	300 ± 10 g	AD model induced by D-gal+AlCl3	No need	KXS,i.g. 2.7, 5.4, 10.8 g/kg/day for 105 days accompanying the model	Normal saline	1. Escape latency in MWM 2. The number of target platform crossings 3. Aβ1–40 plaques level 4. Expression level of Bcl-2 and ChAT	1. *p >* 0.05 2. *p <* 0.01 3. *p <* 0.05 4. *p <* 0.05
Lu et al. (2016)	Wistar rats (male, 12/12)	300–320 g	AD model induced by bilateral hippocampal injection Aβ1–40 with 5 uL	Chloral hydrate (3.5 ml/kg i.p.)	KXS,i.g. 0.72/1.44 g/kg/day for 35 days after the model	Water	1. Ach 2.Glu	1. *p <* 0.01 2. *p <* 0.05
Dang et al. (2009)	SD rats (male and female, 12/11)	190–250 g	Depression model induced by chronic stress	No need	KXS,i.g. 2.7, 0.9, 0.3 g/kg/day for 53 days accompanying the model	Distilled water	1. Sucrose preference index in sucrose preference test 2. Latency for feeding 3. Number of crossing in shuttle box test 4. Total distance in shuttle box test 5. ACTH 6. NE, DA, DOPAC, HVA 7. 5-HT,5-HIAA 8.AchE	1. *p <* 0.01 2. *p <* 0.01 3. *p <* 0.05 4. *p <* 0.01 5. *p <* 0.01 6. *p <* 0.01 7. *p >* 0.05 8. *p <* 0.01
Dong et al. (2016)	Wistar rats (male, 10/10)	180 ± 10 g	Depression model induced by chronic stress	No need	KXS,i.g. 370 mg/kg/day for 3 weeks accompanying the model	Distilled water	1. Sucrose consumption in sucrose preference test 2. Body weight 3. Number of crossing in open field test 4. Rearing count in open field test	1. *p <* 0.05 2. *p <* 0.05 3. *p <* 0.01 4. *p <* 0.05
Dong et al. (2017)	Wistar rats (male, 12/12)	200 ± 10 g	Depression model induced by chronic stress	No need	KXS,i.g. 338, 676 mg/kg/day for 4 weeks accompanying the model	Distilled water	1. Weight 2. Sucrose preference index in sucrose preference test 3. Number of crossing in open field test 4. Rearing count in open field test 5. IL-6 6.TNF-α	1. *p <* 0.01 2. *p <* 0.01 3. *p <* 0.05 4. *p <* 0.05 5. *p >* 0.05 6. *p <* 0.05
Huang et al. (2014)	Wistar rats (male, 12/12)	170–200 g	Depression model induced by chronic stress	3% sodium pentobarbital	KXS,i.g. 65, 130.260 mg/kg/day for 21 days accompanying the model	Distilled water	1. Weight 2. Sucrose consumption in sucrose preference test 3. Score in open field test 4. MT concentration	1. *p >* 0.05 2. *p <* 0.05 3. *p <* 0.05 4. *p <* 0.05
Yan et al. (2016)	SD rats (male, 12/12)	150-180 g	Depression model induced by chronic stress	NR	KXS,i.g. 60.9, 182.7, 548.1mg/kg/day for 6 weeks before the model	Normal saline	1. Sucrose consumption in sucrose preference test 2. Cumulative immobility time in forced swimming test 3. Time spent in central area in open field test 4.Number of rearing in open field test 5. Total distance in open field test 6. NE 7. 5-HT 8.dopamine	1. *p <* 0.01 2. *p <* 0.01 3. *p <* 0.01 4. *p <* 0.01 5. *p <* 0.01 6. *p <* 0.01 7. *p <* 0.01 8. *p <* 0.01
Zhou et al. (2012)	Kunming mice (male, 12/12)	21–30 g	Depression model induced by chronic stress	No need	KXS,i.g. 175, 350, 700, 1,400 mg/kg/day for 3 days before the model	Normal saline	1. Duration of immobility in tail suspension test 2. Duration of immobility in forced swim test 3. 5-HT 4. DA 5.NE	1. *p <* 0.05 2. *p <* 0.05 3. *p <* 0.05 4. *p <* 0.05 5. *p <* 0.05
Dong et al. (2013)	Wistar rats (male, 8/8)	180 ± 10 g	Depression model induced by chronic stress	10% chloral hydrate solution (3.5 ml/kg i.p.)	KXS,i.g. 338, 676 mg/kg/day for 4 weeks accompanying the model	Distilled water	1. Sucrose preference index in sucrose preference test 2. Number of crossing in open field test 3. Rearing times index in open field test 4. Body weight 5. 5-HT,5-HIAA 6. MAO-A, MAO-B	1. *p <* 0.05 2. *p <* 0.05 3. *p <* 0.05 4. *p <* 0.01 5. *p <* 0.01 6. *p >* 0.05
Chu et al. (2016a)	Wistar rats (male, 10/10)	260 ± 20 g	AD model induced by D-gal+AlCl3	Sodium pentobarbital	KXS,i.g. 5.4 g/kg/day for 90 days accompanying the model	Normal saline	1. Escape latency in MWM 2. The number of target platform crossings	1. *p <* 0.01 2. *p <* 0.01
Wang N. et al. (2017)	Wistar rats (male and female, 10/10)	200–240 g	AD model induced by bilateral hippocampal injection Aβ42 with 10 uL	Sodium pentobarbital (50 mg/kg i.p.)	KXS,i.g. 0.54, 1.08 g/kg/day for 21 days after the model	Normal saline	1. Proportion of injured neurons	1.*p <* 0.01
	Wistar rats (male and female,40/40)	200–240 g	AD model induced by bilateral hippocampal injection Aβ42 with 10 uL	Sodium pentobarbital (50 mg/kg i.p.)	KXS,i.g. 0.54, 1.08 g/kg/day for 21 days after the model	Normal saline	1. Aβ42 level 2. hippocampal IDE protein expression 3. IDE mRNA expression	1. *p <* 0.01 2. *p <* 0.01 3. *p >* 0.05
Wang X. J. et al. (2017)	APP/PS1 mice, C57BL/6J mice (male and female, 7/7)	NR	AD model induced by transgenosis	No need	KXS,i.g. 0.65 g/kg/day for 10 months after the model	Distilled water	1. Escape latency in MWM 2. The number of target platform crossings 3. discrimination index in 30 min 4. discrimination index in 24 h 5. Aβ1-42 plaques level	1. *p <* 0.01 2. *p <* 0.01 3. *p <* 0.05 4. *p <* 0.05 5. *p <* 0.05
Zhang et al. (2018)	ICR mice (male and female, 9/8)	25–35 g	AD model induced by lateral ventricle injection Aβ42 with 5 uL	Sodium pentobarbital (45 mg/kg i.p.)	KXS,i.g. 0.15 g/kg/day for 7 days before the model	Normal saline	1. Avoidance time in SDT 2. Error time in SDT	1. *p <* 0.05 2. *p <* 0.05
	ICR mice (male and female, 7/6)	25–35 g	AD model induced by lateral ventricle injection Aβ42 with 5 uL	Sodium pentobarbital (45 mg/kg i.p.)	KXS,i.g. 0.15 g/kg/day for 7 days before the model	Normal saline	1. LTP	1. *P <* 0.05
	ICR mice (male and female,18/15)	25–35 g	AD model induced by lateral ventricle injection Aβ42 with 5 uL	Sodium pentobarbital (45 mg/kg i.p.)	KXS,i.g.0.15g/kg/day for 7 days before the model	Normal saline	1. Number of GluR2 IR cells	1. *p <* 0.01
Huang et al. (2001)	ICR mice (male and female,10/10)	18–21.5 g	AD model induced by SCOP 3 mg/kg	NR	KXS,i.g. 0.1, 0.3 g/kg/day for 7 days before the model	Distilled water	1. NO 2. NOS 3. ChE	1. *p <* 0.01 2. *p <* 0.01 3. *p <* 0.05
Wang et al. (2005)	Kunming mice (male,10/10)	22 ± 2 g	Depression model induced by chronic stress	NR	KXS,i.g. 1.5, 3.6 ml/kg/day for 14 days accompanying the model	Normal saline	1. Duration of immobility in forced swim test 2. GC 3. NE 4. DA 5. 5-HT 6.5-HIAA	1. *p <* 0.05 2. *p <* 0.05 3. *p <* 0.05 4. *p >* 0.05 5. *p <* 0.05 6. *p <* 0.05
Liu Y. M. et al. (2012)	Kunming mice (male,12/12)	22 ± 2 g	Depression model induced by chronic stress	NR	KXS,i.g. 1, 100, 550, 275 mg/kg/day for 7 days before the model	Normal saline	1. Duration of immobility in tail suspension test 2. NE 3. DA 4. 5-HT 5.BDNF	1. *p <* 0.05 2. *p >* 0.05 3. *p <* 0.05 4. *p <* 0.05 5. *p <* 0.01
Shi et al. (2017a)	SAMP8 mice/SAMR1 mice (male,10/10)	24.5–34.2 g/30.2-37.6g	Aging model induced by Gene knockout	No need	KXS,i.g.0.195/0.78 g/kg/day for 8 weeks after the model	Normal saline	1.5-HT 2. 5-HIAA 3. NE 4.DA	1. *p <* 0.01 2. *p <* 0.01 3. *p <* 0.01 4. *p <* 0.01
Shi et al. (2013)	SAMP8 mice/SAMR1 mice (male,10/10)	24.5–34.2 g/30.2–37.6 g	Aging model induced by Gene knockout	No need	KXS,i.g. 0.195/0.78 g/kg/day for 8 weeks after the model	Normal saline	1. TNF-α 2. IL-8 3. β-APP	1. *p <* 0.01 2. *p <* 0.01 3. *p <* 0.01

*Ach, acetylcholine; AchE, Acetyl cholinesterase; ACTH, Adreno cortico tropic hormone; AD, Alzheimer's disease; AGEs, Advanced glycation end products; APP, Amyloid precursor protein; BDNF, brain derived neurotrophic factor; ChAT, choline acetyltransferase; CORT, Corticosterone; CRH, Corticotropin releasing hormone; CRP, Continuous Replenishment Program; DA, Dopamine; DOPAC, Hydroxyphenylacetic acid; GC, Glucocorticoids; Glu, Glucose; GR, Glucocorticoid; GSH-Px, glutathione peroxidase; HVA, Homovanillic acid; Ig, intragastrical administration; KXS, Kaixinsan; LTP, long-term potentiation (LTP); MAO-A, Monoamine oxidase-A; MAO-B, Monoamine oxidase-B; MDA, malondialdehyde; MT, Melatonine; MWM, Morris water maze; NE, Norepinephrine; NOS, Nitric oxide synthase; NR, Not report; SCOP, Scopolamine; SD rats, Sprague Dawley rats; SDT, Step down test; SOD, superoxide dismutase; TNF-α, Tumor Necrosis Factor α; 5-HIAA, 5-hydroxyindoleacetic acid; 5-HT, 5-hydroxytryptamine*.

### Study Quality

Methodological quality scores ranged from 2/10 to 7/10, as shown in Table 2. The mean score was 4.24/10. One (2.6%) study received 7 points, 12 (31.6%) studies received 6 points, 3 (7.9%) studies received 5 points, 6 (15.8%) studies received 4 points, 11 (28.9%) studies received 3 points, and the remaining 5 (13.2%) studies received 2 points. Thirty-three (86.8%) studies included records that were published in peer-reviewed databases or journals, and 5 (13.2%) studies were masters or doctoral theses. Twenty-seven (71.1%) records mentioned control of room temperature. Thirty-seven (97.4%) studies randomly allocated animals to the treatment and control groups. One (2.6%) study used blinded procedures. No (0%) studies mentioned blind induction of the model, or calculations to determine sample size. Twenty-nine (76.3%) studies used anesthetics without significant intrinsic neuroprotective activity. Five (13.2%) studies used appropriate animal models. Eighteen (47.4%) studies complied with animal protection law. Eleven (28.9%) studies declared no potential conflicts of interests.

**Table 2:** Risk of bias of the induced studies.

**Study**	**A**	**B**	**C**	**D**	**E**	**F**	**G**	**H**	**I**	**J**	**Total**
Bian et al. (2000)	√		√								2
Shang (2003)		√	√								2
Bian et al. (1999)	√					√	√				3
Zhou et al. (2008)	√		√			√					3
Gao et al. (2010)	√	√	√								3
Li et al. (2016)	√		√			√					3
Xu and Jiang (2017)	√		√			√					3
Zhong (2005)		√	√			√					3
Shi et al. (2017b)	√	√	√			√	√		√		6
Huang et al. (1999)	√		√								2
Huang et al. (1998)	√		√			√	√				4
Dang (2008)		√	√			√			√		4
Zhang et al. (2016)	√	√	√			√					4
Wang et al. (2007)	√	√	√			√					4
Liu M. et al. (2012)	√		√			√					3
Liu W. W. et al. (2015)	√		√			√					3
Fonarow (2016)	√	√	√			√					4
Zhang et al. (2018)		√	√			√					3
Dou (2017)		√	√			√					3
Xu et al. (2019)	√	√	√			√			√	√	6
Chu et al. (2016b)	√	√	√			√			√	√	6
Lu et al. (2016)	√	√	√			√			√	√	6
Dang et al. (2009)	√	√	√			√			√		5
Dong et al. (2016)	√	√	√			√			√	√	6
Dong et al. (2017)	√	√	√			√			√	√	6
Huang et al. (2014)	√	√	√			√			√	√	6
Yan et al. (2016)	√	√	√						√	√	5
Zhou et al. (2012)	√	√	√		√	√			√	√	7
Dong et al. (2013)	√	√	√						√		4
Chu et al. (2016a)	√	√	√			√			√		5
Wang N. et al. (2017)	√	√	√			√				√	6
Wang X. J. et al. (2017)	√	√	√			√			√	√	6
Zhang et al. (2018)	√	√	√			√			√	√	6
Huang et al. (2001)	√		√								2
Wang et al. (2005)	√		√								2
Liu Y. M. et al. (2012)	√	√	√								3
Shi et al. (2017b)	√	√	√			√	√		√		6
Shi et al. (2013)	√	√	√			√	√		√		6

*Studies fulfilling the criteria of: A, peer reviewed publication; B, control of temperature; C, random allocation to treatment or control; D, blinded induction of model; E, blinded assessment of outcome; F, use of anesthetic without significant intrinsic neuroprotective activity; G, appropriate animal model; H, sample size calculation; I, compliance with animal welfare regulations; J, statement of potential conflict of interests*.

### Effectiveness

**Figure 2:**
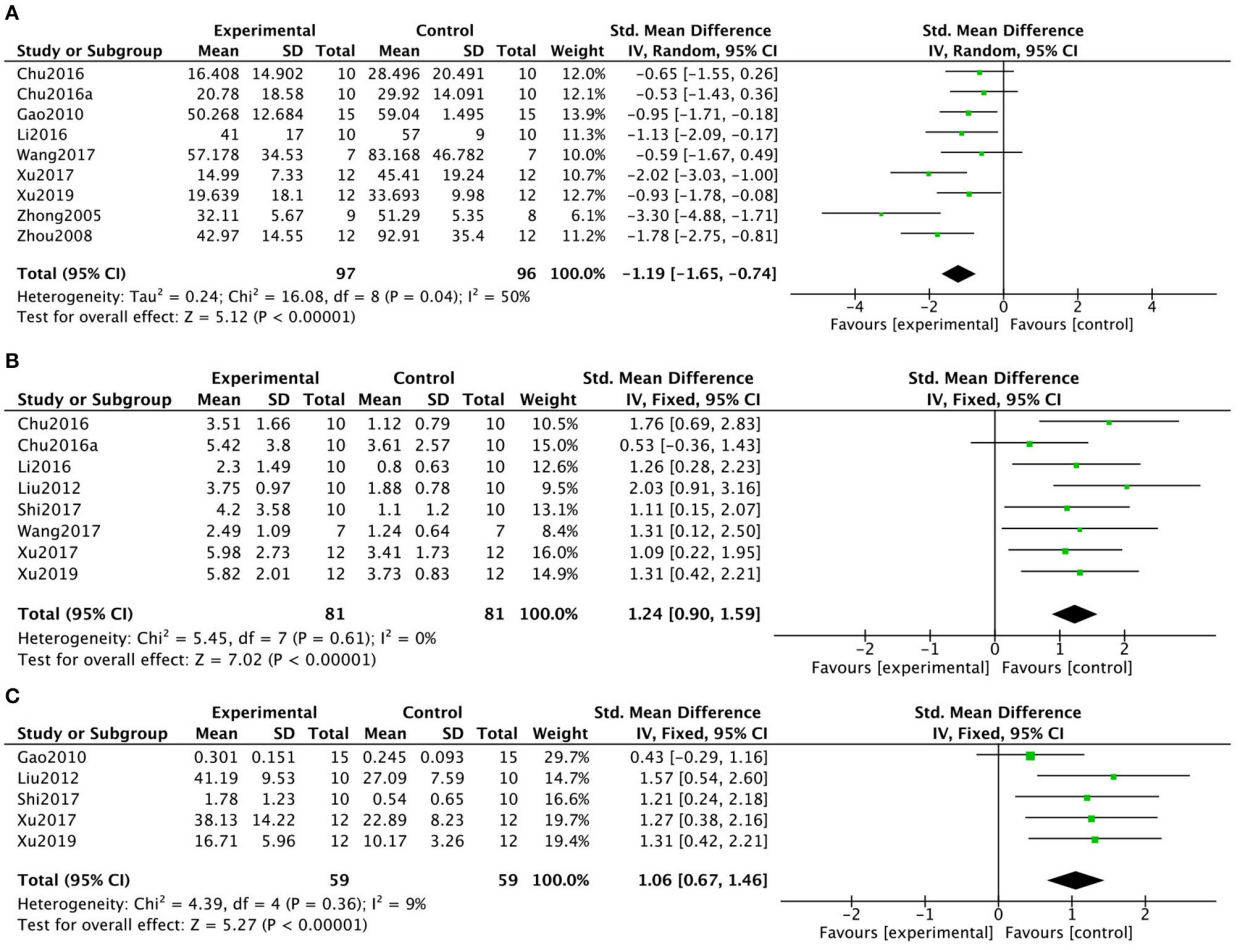
Forest plot showing that KXS treatment decreased escape latency **(A)**, increased the number of target platform crossings **(B)**, and increased time spent on the target quadrants **(C)** in the Morris water maze test compared with the Control group.

As an indicator of cognitive function, 9 (23.7%) studies (Zhong, 2005; Zhou et al., 2008; Gao et al., 2010; Chu et al., 2016a,b; Li et al., 2016; Wang X. J. et al., 2017; Xu and Jiang, 2017; Xu et al., 2019) measured escape latency in the MWM. The pooled data showed that KXS significantly decreased escape latency in the MWM (*P* < 0.00001; SMD = −1.19, 95% CI [−1.65, −0.74]; Heterogeneity: χ2 = 16.08, df = 8 (*P* = 0.04); *I*^2^ = 50%, Figure 2A). Eight (21.1%) studies (Liu M. et al., 2012; Chu et al., 2016a,b; Li et al., 2016; Shi et al., 2017b; Wang X. J. et al., 2017; Xu and Jiang, 2017; Xu et al., 2019) reported the number of target platform crossings in the MWM as an indicator of cognitive function. The pooled data showed a significant difference between the KXS treatment groups and the control groups (*P* < 0.00001; SMD = 1.24, 95% CI [0.90, 1.59]; Heterogeneity: χ2 = 5.45, df = 7 (*P* = 0.61); *I*^2^ = 0%, Figure 2B). Five (13.2%) studies (Gao et al., 2010; Liu M. et al., 2012; Shi et al., 2017b; Xu and Jiang, 2017; Xu et al., 2019) reported the length of time spent in the target quadrants as an indicator of memory function. The pooled data showed that KXS treatment resulted in a marked difference in the length of time spent in the target quadrant between the KXS and control groups (*P* < 0.00001; SMD = 1.06, 95% CI [0.67, 1.46]; Heterogeneity: χ2 = 4.39, df = 4 (*P* = 0.36); *I*^2^ = 9%, Figure 2C).

**Figure 3:**
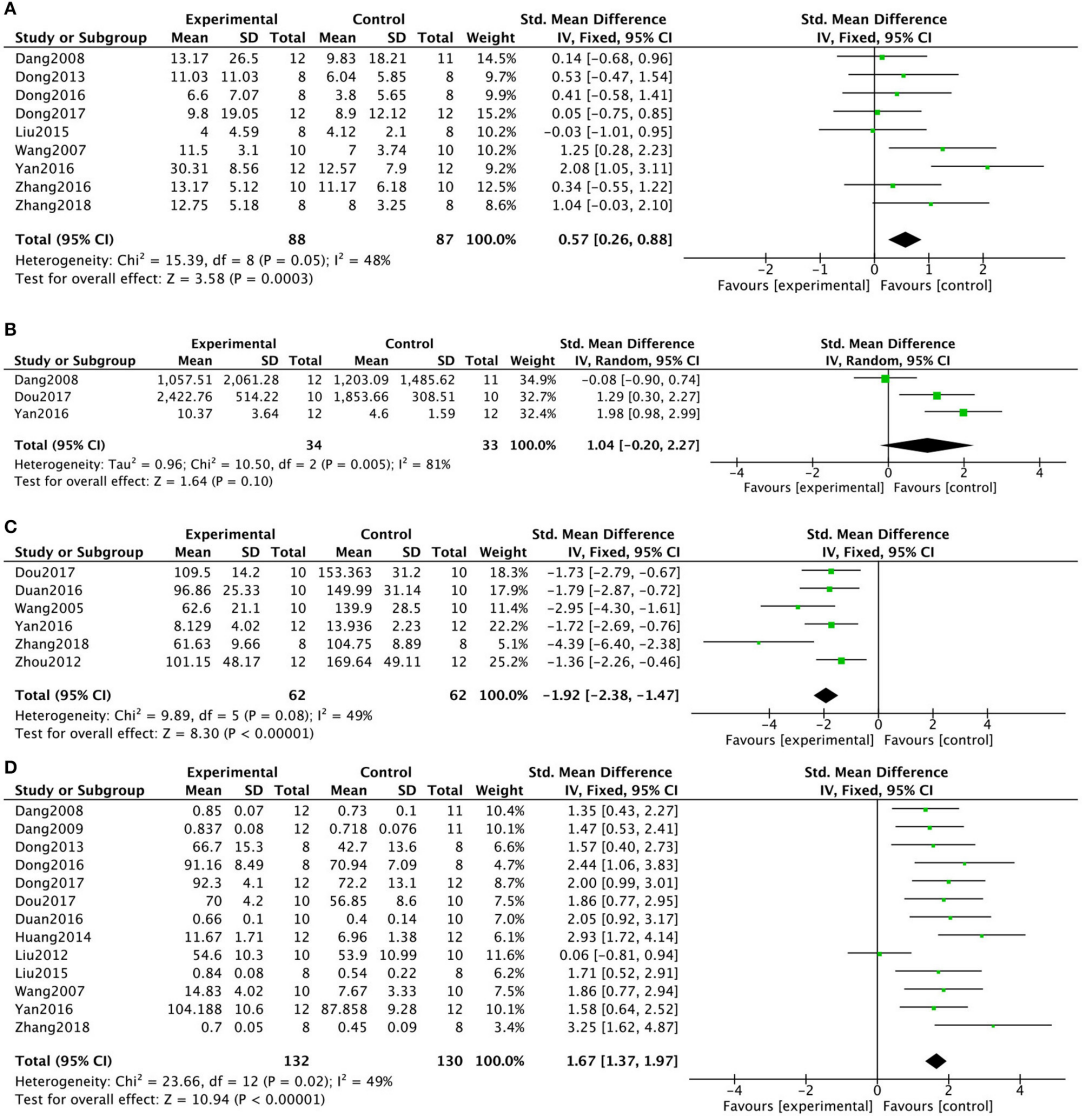
Forest plot showing that KXS increased the number of rearing events **(A)** and total distance **(B)** in the open-field test, decreased the duration of immobility in the forced swimming test **(C)**, and increased sucrose consumption in the sucrose preference test **(D)** compared with the Control group.

As an indicator of depression, 9 (23.7%) studies (Wang et al., 2007; Dang, 2008; Dong et al., 2013, 2016, 2017; Liu W. W. et al., 2015; Yan et al., 2016; Zhang et al., 2016; Zhang, 2018) reported the number of rearing events in the open-field test. Kaixinsan induced a marked increase in the number of rearing events in the open-field test compared with that in the control group (*P* = 0.0003; SMD = 0.57, 95% CI [0.26, 0.88]; Heterogeneity: χ2 = 15.39, df = 9 (*P* = 0.05); *I*^2^ = 48%, Figure 3A). Three (7.9%) studies (Dang, 2008; Yan et al., 2016; Dou, 2017) reported total distance in the open-field test. The pooled data showed no significant difference between the KXS treatment groups and the control groups (*P* = 0.10; SMD = 1.04, 95% CI [−0.20, 2.27]; Heterogeneity: χ2 = 10.50, df = 2 (*P* = 0.005); *I*^2^ = 81%, Figure 3B). When only studies that used male animals were included, a meta-analysis of 2 studies (Yan et al., 2016; Dou, 2017) showed a significant difference between the KXS groups and the control groups, with the *I*^2^ value dropping from 81 to 0% (*P* < 0.00001; SMD = 1.63, 95% CI [0.92, 2.33]; Heterogeneity: χ2 = 0.94, df = 1 (*P* = 0.33); *I*^2^ = 0%). Six (15.8%) studies (Wang et al., 2005; Zhou et al., 2012; Fonarow, 2016; Yan et al., 2016; Dou, 2017; Zhang, 2018) evaluated duration of immobility in the forced swim test. The pooled data showed that KXS treatment resulted in a marked drop in the duration of immobility in the forced swimming test compared with that in the control groups (*P* < 0.00001; SMD = -1.92, 95% CI [−2.38, −1.47]; Heterogeneity: χ2 = 9.89, df = 5 (*P* = 0.08); *I*^2^ = 49%, Figure 3C). Thirteen (34.2%) studies (Wang et al., 2007; Dang, 2008; Dang et al., 2009; Liu M. et al., 2012; Dong et al., 2013, 2016, 2017; Huang et al., 2014; Liu W. W. et al., 2015; Fonarow, 2016; Yan et al., 2016; Dou, 2017; Zhang, 2018) evaluated sucrose consumption or sucrose preference index in the sucrose preference test. Treatment with KXS resulted in significantly different sucrose consumption or sucrose preference than that in the control group (*P* < 0.00001; SMD = 1.67, 95% CI [1.37, 1.97]; Heterogeneity: χ2 = 23.66, df = 12 (*P* = 0.02); *I*^2^ = 49%, Figure 3D).

**Figure 4:**
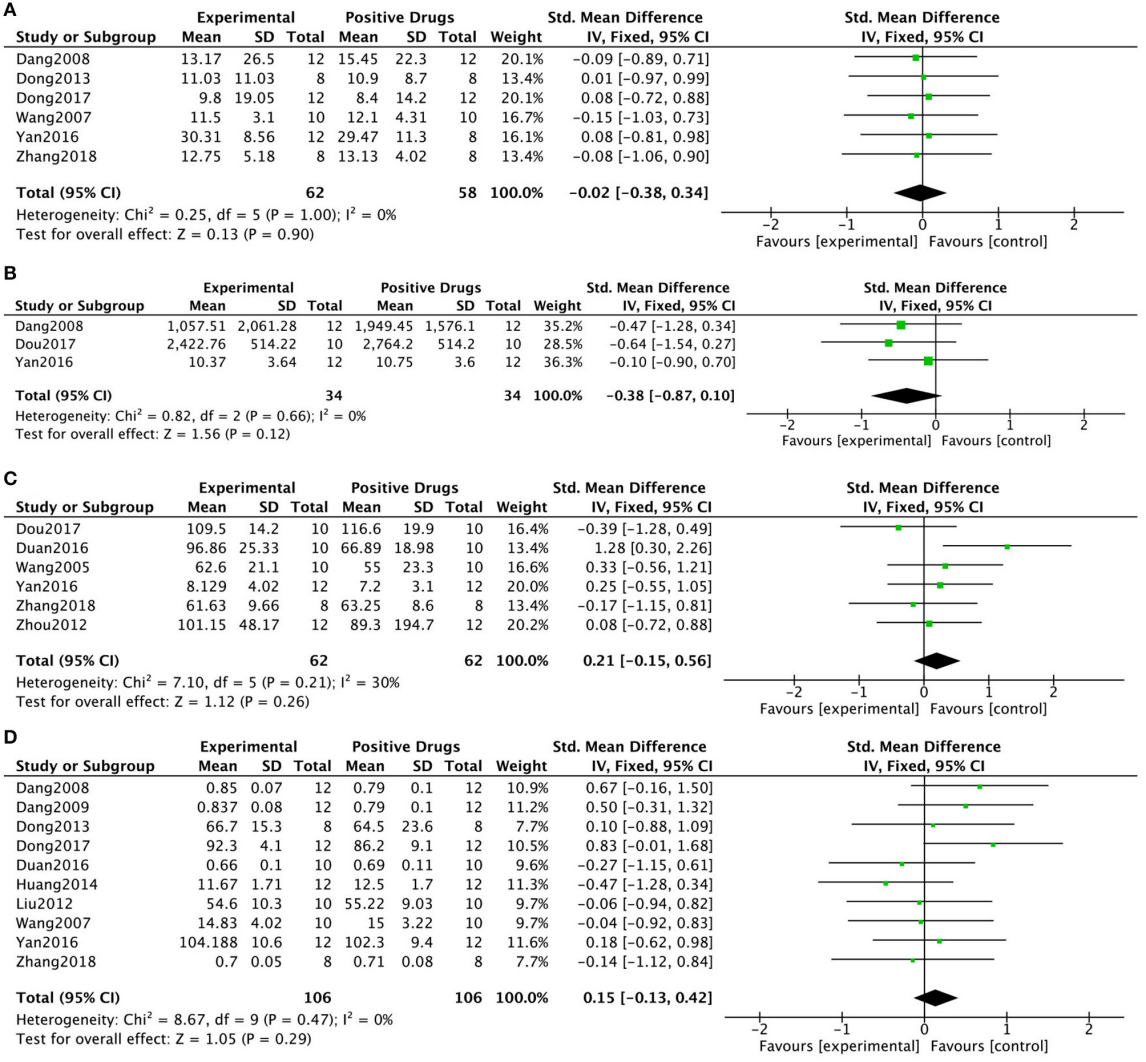
Forest plot showing that KXS showed no significant difference compared with other drugs on the number of rearing events **(A)** and total distance **(B)** in the open-field test; There were no differences in duration of immobility in the forced swimming test **(C)**; There were no differences in sucrose consumption in the sucrose preference test **(D)**.

Six (15.8%) studies (Wang et al., 2007; Dang, 2008; Dong et al., 2013, 2017; Yan et al., 2016; Zhang, 2018) that compared KXS with positive drug treatments reported the number of rearing events in the open-field test. There were no significant differences between the experimental groups and the drug groups (*P* = 0.90; SMD = −0.02, 95% CI [−0.38, 0.34]; Heterogeneity: χ2 = 0.25, df = 5 (*P* = 1.00); *I*^2^ = 0%, Figure 4A). Three (7.9%) studies (Dang, 2008; Yan et al., 2016; Dou, 2017) reported the total distance in the open-field test. The pooled data showed no significant differences between the KXS treatment groups and the positive drug groups (*P* = 0.12; SMD = −0.38, 95% CI [−0.87, 0.10]; Heterogeneity: χ2 = 0.82, df = 2 (*P* = 0.66); *I*^2^ = 0%, Figure 4B). Six (15.8%) studies (Wang et al., 2005; Zhou et al., 2012; Fonarow, 2016; Yan et al., 2016; Dou, 2017; Zhang, 2018) reported the duration of immobility in the forced swim test. The pooled data showed that there were no differences between the KXS treatment groups and the positive drug groups (*P* = 0.26; SMD = 0.21, 95% CI [−0.15, 0.56]; Heterogeneity: χ2 = 7.10, df = 5 (*P* = 0.21); *I*^2^ = 30%, Figure 4C). Ten (26.3%) studies (Wang et al., 2007; Dang, 2008; Dang et al., 2009; Liu M. et al., 2012; Dong et al., 2013, 2017; Huang et al., 2014; Fonarow, 2016; Yan et al., 2016; Zhang, 2018) reported sucrose consumption or sucrose preference index in the sucrose preference test. There were no significant differences between the KXS treatment groups and the positive drug groups (*P* = 0.29; SMD = 0.15, 95% CI [−0.13, 0.42]; Heterogeneity: χ2 = 8.67, df = 9 (*P* = 0.47); *I*^2^ = 0%, Figure 4D).

### Mechanisms of Kaixinsan for Cognitive Impairment and Depression

**Figure 5:**
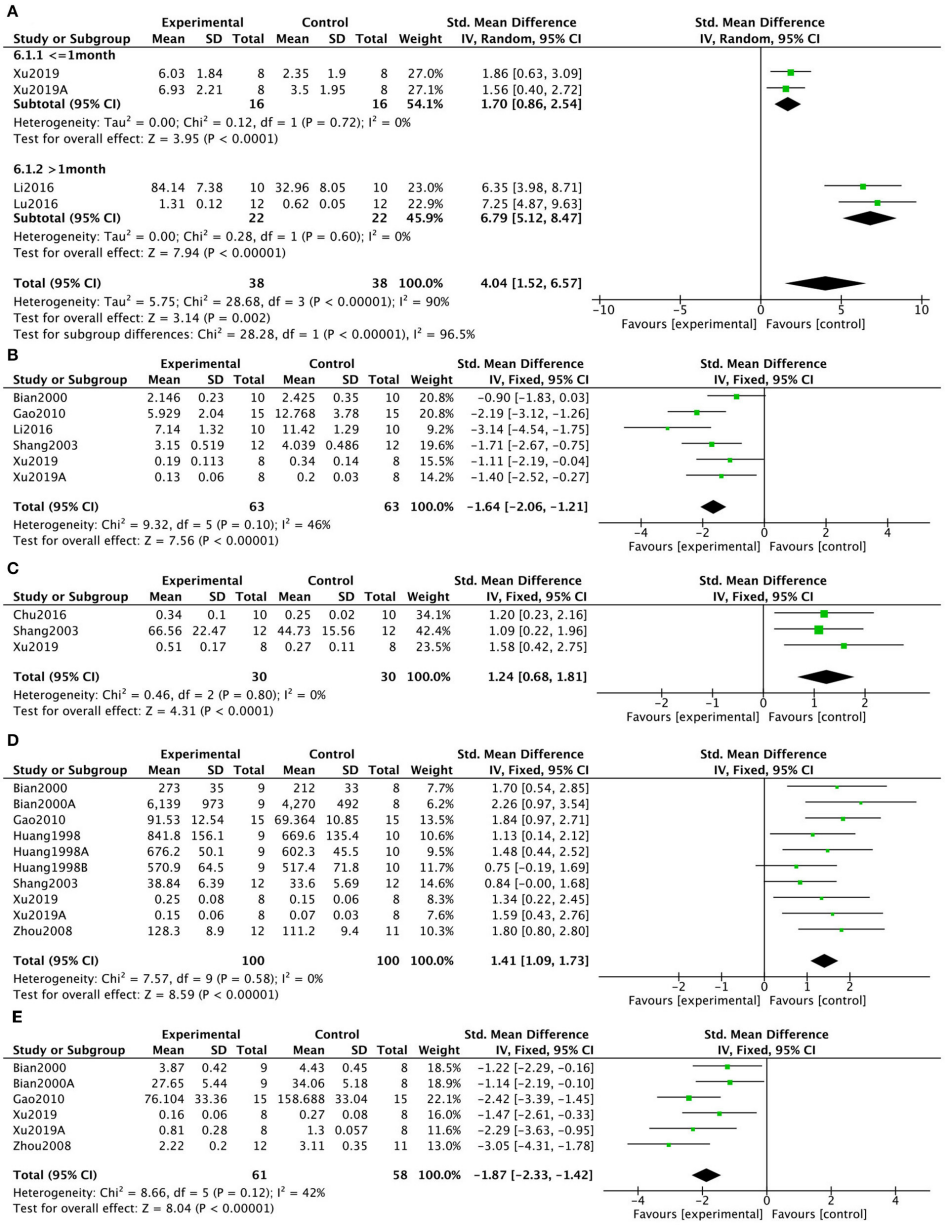
Forest plot showing that KXS increased ACh levels **(A)**, decreased AchE activity **(B)**, increased ChAT activity **(C)**, increased SOD levels **(D)**, and decreased MDA levels **(E)** compared with controls in AD models.

Pooled data from 4 studies in 3 (7.9%) manuscripts (Li et al., 2016; Lu et al., 2016; Xu et al., 2019) showed that KXS significantly increased acetylcholine activity (*P* < 0.00001; SMD = 4.04, 95% CI [1.52, 6.57]; Heterogeneity: χ2 = 28.68, df = 3 (*P* < 0.00001); *I*^2^ = 90%, Figure 5A). To identify potential sources of heterogeneity, subgroup analysis of ACh activity was performed based on the duration of treatment. The results showed that longer periods of KXS treatment resulted in larger effect sizes (SMD = 6.79 vs. SMD = 1.70). Six studies in 5 (13.2%) manuscripts (Bian et al., 2000; Shang, 2003; Gao et al., 2010; Li et al., 2016; Xu et al., 2019) showed increased acetylcholinesterase (AchE) activity in response to KXS (*P* < 0.00001; SMD = −1.64, 95% CI [−2.06, −1.21]; Heterogeneity: χ2 = 9.32, df = 5 (*P* = 0.10); *I*^2^ = 46%, Figure 5B). Three (7.9%) studies (Shang, 2003; Chu et al., 2016b; Xu et al., 2019) showed increased ChAT activity in response to KXS (*P* < 0.00001; SMD = 1.24, 95% CI [0.68, 1.81]; Heterogeneity: χ2 = 0.46, df = 2 (*P* = 0.80); *I*^2^ = 0%, Figure 5C). Ten studies in 6 (15.8%) manuscripts (Huang et al., 1998; Bian et al., 2000; Shang, 2003; Zhou et al., 2008; Gao et al., 2010; Xu et al., 2019) showed increased levels of SOD in response to KXS (*P* < 0.00001; SMD = 1.41, 95% CI [1.09, 1.73]; Heterogeneity: χ2 = 7.57, df = 10 (*P* = 0.58); *I*^2^ = 0%, Figure 5D). Six studies in 4 (10.5%) manuscripts (Bian et al., 2000; Zhou et al., 2008; Gao et al., 2010; Xu et al., 2019) showed decreased levels of MDA in response to KXS (*P* < 0.00001; SMD = −1.87, 95% CI [−2.33, −1.42]; Heterogeneity: χ2 = 8.66, df = 5 (*P* = 0.12); *I*^2^ = 42%, Figure 5E).

**Figure 6:**
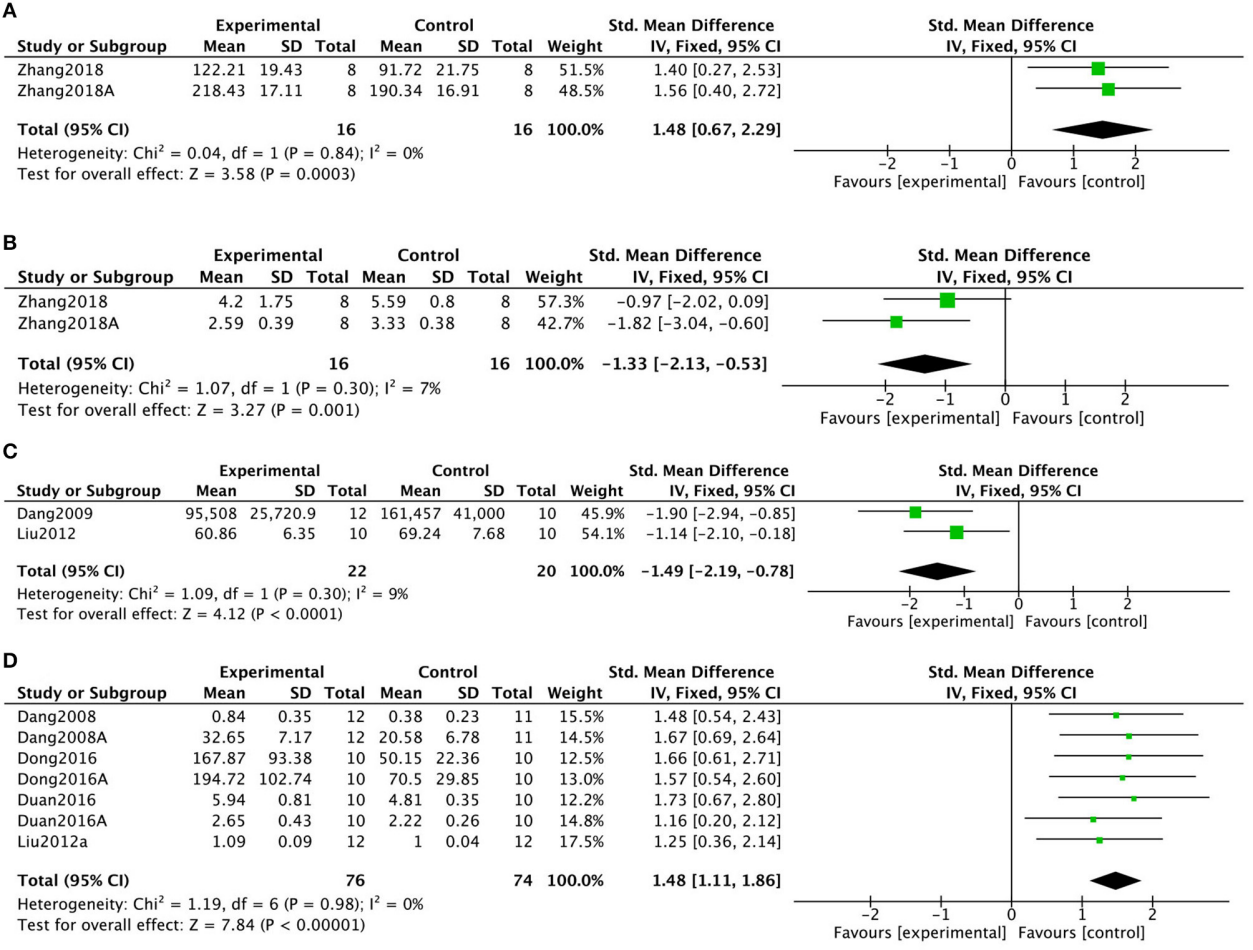
Forest plot showing that KXS increased SOD levels **(A)**, decreased MDA levels **(B)**, decreased AchE activity **(C)**, and increased BDNF levels **(D)** compared with controls in depression models.

**Figure 7:**
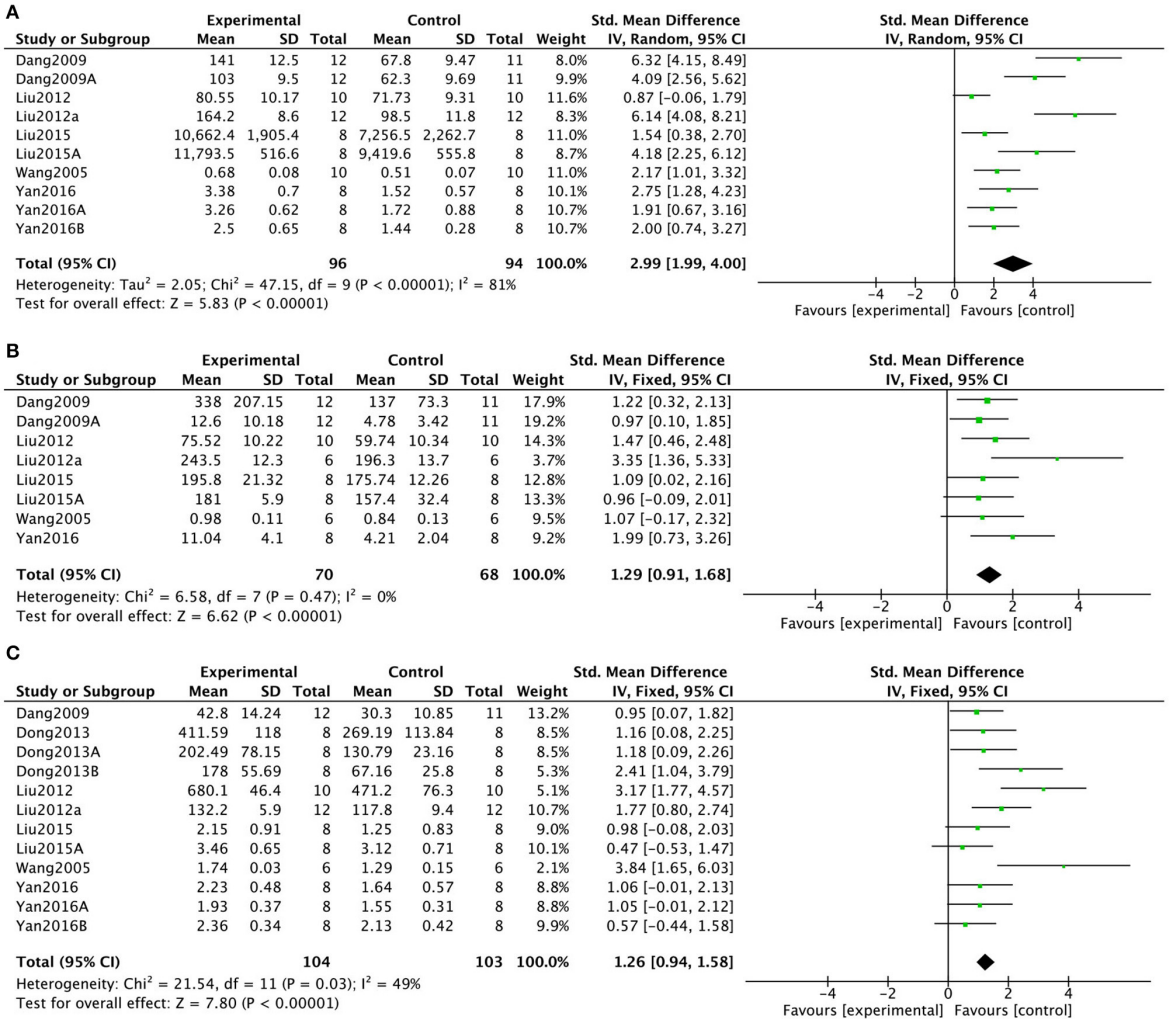
Forest plot showing that KXS NE **(A)**, DA **(B)**, and 5-HT **(C)** levels compared with controls in depression models.

To determine the mechanisms of KXS in treatment of depression, 2 studies in 1 (2.6%) article (Zhang, 2018) showed that KXS increased superoxide dismutase levels (*P* = 0.0003; SMD = 1.48, 95% CI [0.67, 2.29]; Heterogeneity: χ2 = 0.04, df = 1 (*P* = 0.84); *I*^2^ = 0%, Figure 6A). In addition, 2 studies in 1 (2.6%) article (Zhang, 2018) showed that KXS decreased malondialdehyde levels (*P* = 0.001; SMD = −1.33, 95% CI [−2.13, −0.53]; Heterogeneity: χ2 = 1.07, df = 1 (*P* = 0.30); *I*^2^ = 7%, Figure 6B). Two (5.3%) studies (Dang et al., 2009; Liu M. et al., 2012) showed that KXS treatment resulted in decreased AchE activity (*P* < 0.0001; SMD = −1.49, 95% CI [−2.19, −0.78]; Heterogeneity: χ2 = 1.09, df = 1 (*P* = 0.30); *I*^2^ = 9%, Figure 6C). Six studies in 4 (10.5%) articles (Dang, 2008; Liu Y. M. et al., 2012; Dong et al., 2016; Fonarow, 2016) showed decreased levels of brain-derived neurotrophic factor (BDNF) in response to KXS treatment (*P* < 0.00001; SMD = 1.48, 95% CI [1.11, 1.86]; Heterogeneity: χ2 = 1.19, df = 6 (*P* = 0.98); *I*^2^ = 0%, Figure 6D). Ten studies in 6 (15.8%) articles (Wang et al., 2005; Dang et al., 2009; Liu M. et al., 2012; Liu Y. M. et al., 2012; Liu W. W. et al., 2015; Yan et al., 2016) showed increased levels of NE in response to KXS (*P* < 0.00001; SMD = 2.99, 95% CI [1.99, 4.00]; Heterogeneity: χ2 = 47.15, df = 9 (*P* < 0.00001); *I*^2^ = 81%, Figure 7A). Eight studies in 6 (15.8%) articles (Wang et al., 2005; Dang et al., 2009; Liu M. et al., 2012; Liu Y. M. et al., 2012; Liu W. W. et al., 2015; Yan et al., 2016) showed increased levels of DA in response to KXS (*P* < 0.00001; SMD = 1.29, 95% CI [0.91, 1.68]; Heterogeneity: χ2 = 6.58, df = 7 (*P* = 0.47); *I*^2^ = 0%, Figure 7B). Twelve studies in 7 (18.4%) articles (Wang et al., 2005; Dang et al., 2009; Liu M. et al., 2012; Liu Y. M. et al., 2012; Dong et al., 2013; Liu W. W. et al., 2015; Yan et al., 2016) showed increased concentrations of 5-HT in response to KXS (*P* < 0.00001; SMD = 1.26, 95% CI [0.94, 1.58]; Heterogeneity: χ2 = 21.54, df = 11 (*P* = 0.03); *I*^2^ = 49%, Figure 7C).

## Discussion

### Summary of Evidence

This is the first preclinical systematic review of KXS for cognitive impairment and depression based on 38 included studies with 1,050 animals. The methodological quality score was a mean of 4.24. The results demonstrated that KXS could significantly ameliorate both the behavioral performance of cognitive impairment and depression in animal models. The former included shortening the escape latency, decreasing the time spent on the target quadrants and increasing the number of target platform crossings in MWM, and the latter included raising the number of rearing in open-field test, decreasing the duration of immobility in forced swimming test and increasing the sucrose consumption or sucrose preference index in sucrose preference test. The meta-analysis of biochemical indicators illustrated that possible mechanisms of KXS include improvement of cognitive function via antioxidant, anti-inflammatory, antiapoptotic, neuroprotection, and synapse protection in cognitive impairment models, and antidepression effects through HPA axis regulation, antioxidant, anti-inflammatory, synapse protection and nervous protection in depression models. The findings of present study indicated that KXS exerted a consistent effect on improving the memory defects and depression symptoms in multiple animal models, indicating the therapeutic potential of KXS for treating AD-related BPSD.

### Methodological Considerations

Currently, two major quality assessment tools are typically applied in preclinical systematic reviews: The CAMARADES checklist and the SYRCLE's risk of bias tool. The CAMARADES checklist was designed as a tool to evaluate the methodological quality of interventional studies using stroke models. Subsequently, this checklist was used to evaluate other neurological diseases because the confounding factors are similar. The SYRCLE's risk of bias tool for animal studies originated from the Risk of Bias tool by Cochrane Collaboration (Hooijmans et al., 2014).

In this study, we used the CAMARADES checklist to assess quality because AD and depression are neurological disorders. The methodological quality score of the included studies were generally intermediate. The main concerns were as follows. Despite almost all statement randomly, no studies mentioned the detailed methods of random allocation in the present study. Randomization is a critical step to reduce selection bias, which ensures that comparisons are unbiased and that findings of studies are valid (Fonarow, 2016). Animal studies that do not use randomization are more likely to obtain positive results (Bebarta et al., 2003). However, randomization is often conducted poorly and reported incompletely (Doig and Simpson, 2005). According to the ARRIVE guidelines (Kilkenny et al., 2010), randomization procedures should be conducted appropriately, and reported in detail.

Results are more objective when using blind assessment (Schulz et al., 1995). Furthermore, the variability of observer make sense in outcomes (Bebarta et al., 2003). However, only a few studies use appropriate blinding procedures. Failure to blind studies may result in potential bias during modeling and outcome assessment. The ARRIVE guidelines (Kilkenny et al., 2010) state that steps taken to reduce the bias, such as blinding, should be detailed within manuscripts.

Calculation of sample size is important to ensure that enough participants are included to appropriately determine statistical significance (Khaled Fahim and Negida, 2018). Small sample sizes can result in not capturing true effect sizes, while inappropriately large sample sizes can be associated with ethical issues (Fitts, 2011). However, descriptions of how sample sizes are chosen are often inadequate (Baker et al., 2014). None of the included studies detailed how sample size was determined. The ARRIVE guidelines (Kilkenny et al., 2010) stated that the method by which sample size was determined should be appropriately detailed.

Seven studies used sodium pentobarbital as an anesthetic, and 2 studies used chloral hydrate. Pentobarbital is a commonly used barbiturate in animal studies. However, it must be used carefully because it can induce respiratory distress and deep sleep (Feustel et al., 1981; Warner et al., 1996). Furthermore, it is unknown whether barbiturates induce neuroprotective effects (Zwerus and Absalom, 2015). Although chloral hydrate has not been shown to induce neuroprotection in rats (Ozden and Isenmann, 2004), it has been shown to induce systemic toxicity in rodents (Huske et al., 2016). In addition, chloral hydrate may also be a carcinogen in rodents, which is an ethical issue (Maud et al., 2014). It is essential to select appropriate anesthetics in neurological studies.

### Implications

Preclinical systematic review is a scientific approach to synthesize preclinical evidence with the goal of informing future studies. Preclinical systematic review provides an ethical approach and can increase sample sizes without increasing use of resources or number of animals. This systematic review synthesized preclinical evidence for KXS as a treatment for AD and depression. The results of our meta-analysis showed that KXS improved AD and depression symptoms in rats and mice. The results of the present study suggested that KXS may be a novel therapeutic agent for treatment of the behavioral and psychological symptoms of Alzheimer's dementia in preclinical and clinical studies.

Because AD is a chronic disease, it is important to assess the long-term effects and safety of KXS (Wimo, 2015). However, the progressive nature of AD makes long-term assessment of treatment strategies difficult (Rogers and Friedhoff, 1998). Thus, studies that evaluate the long-term efficacy and safety of KXS are needed.

The blood–brain barrier (BBB) is a physiological barrier that protects the central nervous system from harmful chemicals and peripheral biomolecules. In addition, the BBB prevents many drugs from entering the brain, resulting in low bioavailability in the brain and reduced pharmacological effects. Many drugs have failed to translate to clinical use despite being effective for mitigation of CNS disorders because they are unable to cross the human BBB (Patel and Patel, 2017). Although the present systematic review provides evidence that KXS can improve cognitive impairment and depression, the mechanisms of KXS crosses the BBB is unknown. Further studies should evaluate KXS and its active ingredients crossing the BBB, and should focus on development of formulations that maximize brain bioavailability of KXS.

Animal models are essential for human disease research. Appropriate animal models should accurately reproduce the pathophysiology of human disease. There are 3 major types of AD models currently used: spontaneous models, chemically induced models, and transgenic animal models (Neha et al., 2014). Aging models and the SAMP8 mouse model were included as spontaneous models in our systematic review. Spontaneous models can accurately mimic human AD, but these studies are expensive and time-consuming. Chemically induced models were included in the present study, such as a scopolamine-induced model, an Aβ infusion-induced model, a D-gal-induced model, and an AlCl3-induced model. These models are widely used because they are easy to implement, and are relatively inexpensive. However, these models do not accurately mimic the pathophysiology of AD (Esquerda-Canals et al., 2017). APP/PS1 mice are a transgenic animal model included in our study. Transgenic animal models are frequently used due to advances in technology and the existence of well-established procedures (Esquerda-Canals et al., 2017). However, transgenic models are difficult to implement in rats (Do Carmo and Cuello, 2013).

The animal models of depression included in this systematic review were all induced by chronic stress, which is one of the most valid approaches for modeling depression (Willner, 1984). Depression is a disease with complex and varied etiology, and only one-fourth of patients develop depression due to stress (Willner, 1984). Because social stress results in varied responses, it is of great importance to carefully evaluate associations between preclinical and clinical studies. Furthermore, common conditions that induce depressive behaviors in humans and animals should be considered when attempting to translate preclinical evidence to the clinic (McArthur and Borsini, 2006).

There is a lack of systematic research on models that mimic the combination of AD and depression. The ability of depression models to reproduce the pathophysiology of AD-related depression needs further study. Future studies should aim to develop better models to study BPSD.

Alzheimer's disease is a progressive disease that results in disability and death. Behavioral and psychological symptoms of dementia are a set of behaviors and neuropsychiatric symptoms associated with AD. Depression is one of the most common BPSDs associated with AD (Preuss et al., 2016), and correlates with accelerated AD-related cognitive impairment (Bassuk et al., 1998), increased mortality (Verkaik et al., 2007), and increased incidence of depression in caregivers (Barca et al., 2009). As a consequence, AD-related depression results in reduced quality of life of patients and increased social burden. Currently, the primary treatments for AD only control symptoms, but do not halt or cure AD. Behavioral and psychological symptoms of dementia are a diverse set of symptoms, which has prevented development of an appropriate single treatment approach for BPSD. Alzheimer's disease and depression are two conditions that often exist simultaneously in elderly individuals, they share many common symptoms (Novais and Starkstein, 2015), and both are associated with neurobiological changes such as cortical atrophy, limbic atrophy, and white matter lesions (Bennett and Thomas, 2014). The components of KXS act synergistically and interact with multiple targets, which may result in better treatment of AD and AD-related psychological symptoms. This systematic review synthesized preclinical evidence, and showed that KXS may be a promising therapeutic agent for treatment of AD and depression. However, the mechanisms by which KXS acts upon AD and depression are not clear.

### Mechanisms

**Figure 8:**
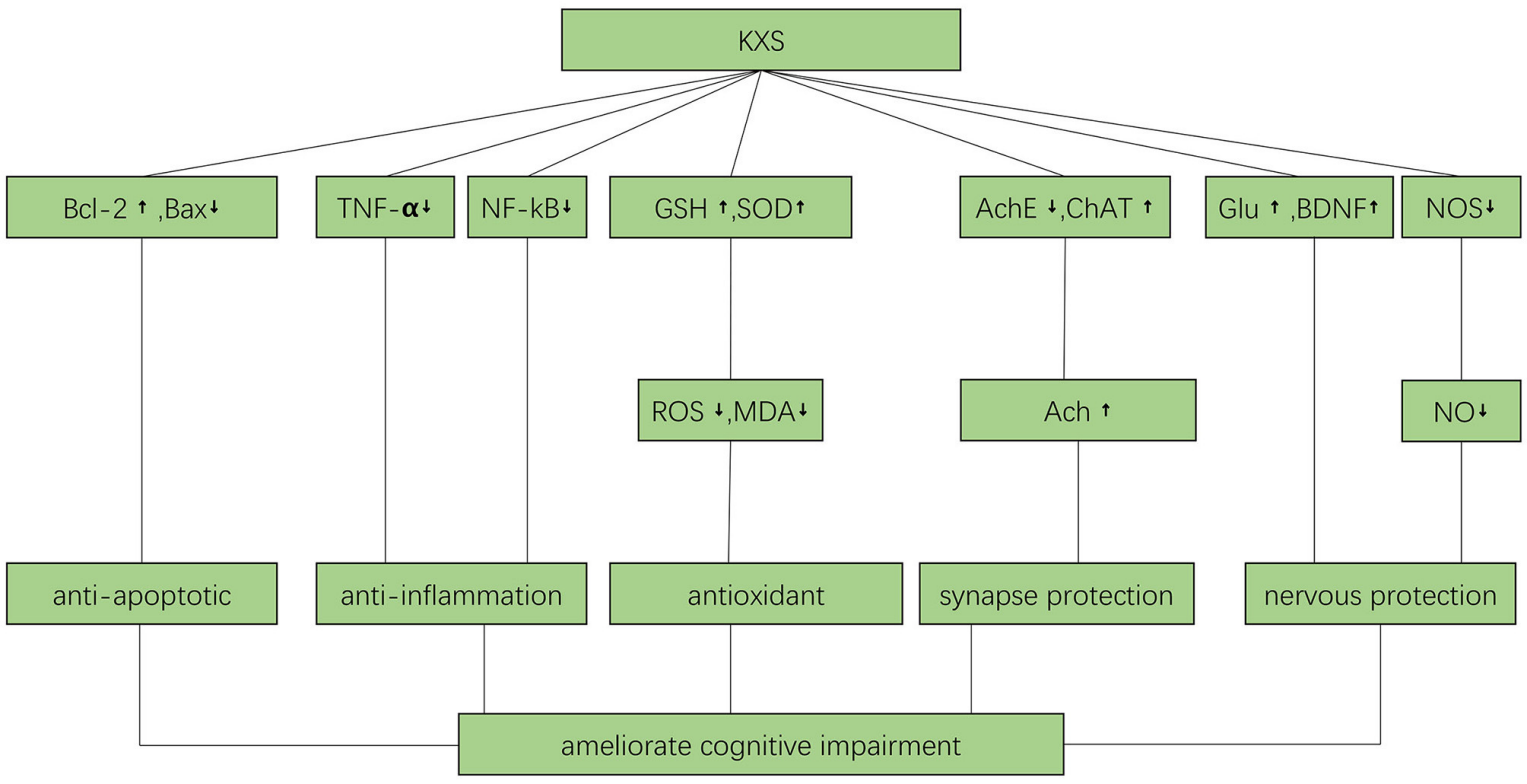
The possible mechanisms by which KXS may improve cognitive function. Ach, acetylcholine; AchE, Acetyl cholinesterase; BDNF, brain derived neurotrophic factor; ChAT, choline acetyltransferase; Glu, Glucose; GSH, glutathione; MDA, malondialdehyde; NF-kB, nuclear factor-k-gene binding; NO, Nitric oxide; NOS, Nitric oxide synthase; ROS, reactive oxygen species; SOD, superoxide dismutase; TNF-α, Tumor Necrosis Factor α.

**Figure 9:**
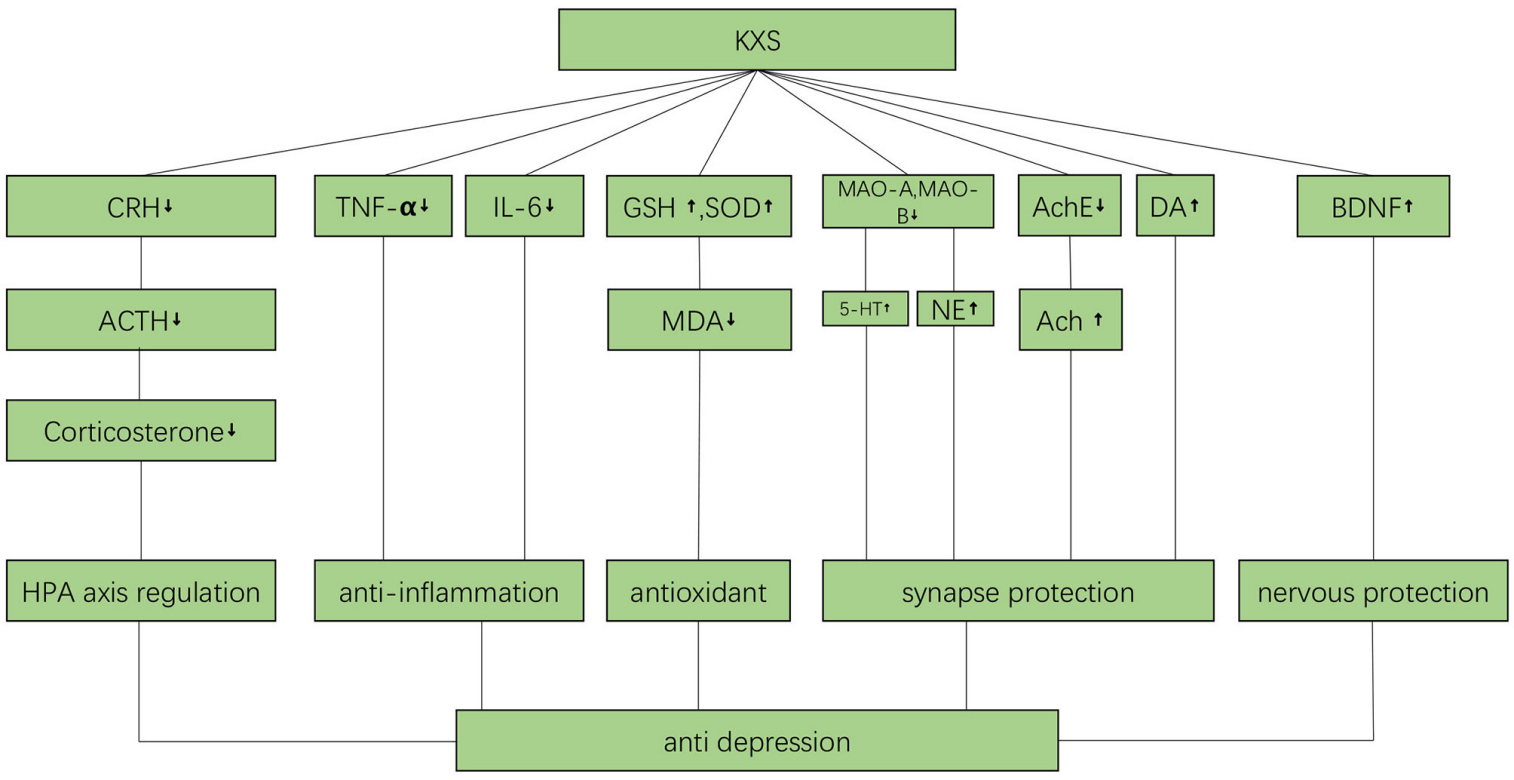
The possible mechanisms by which KXS may ameliorate depression. Ach, acetylcholine; AchE, Acetyl cholinesterase; ACTH, Adreno cortico tropic hormone; BDNF, brain derived neurotrophic factor; CRH, Corticotropin releasing hormone; DA, Dopamine; GSH, glutathione; IL-6, interleukin-6; MAO-A, Monoamine oxidase-A; MAO-B, Monoamine oxidase-B; MDA, malondialdehyde; NE, Norepinephrine; TNF-α, Tumor Necrosis Factor α; 5-HT, 5-hydroxytryptamine.

The mechanisms by which KXS ameliorated cognitive impairment were as follows (Figure 8). Kaixinsan may play a neurotrophic role by increasing glutamate (Glu) and brain-derived neurotrophic factor (BDNF) levels, and reducing nitric oxide-induced neurotoxicity via down-regulation of nitric oxide synthase activity. Kaixinsan also up-regulated the expression of Bcl-2 and down-regulated the expression of Bax, resulting in anti-apoptotic effects. Furthermore, KXS decreased TNF-α and NF-κB levels, resulting in anti-inflammatory effects. In addition, KXS decreased ROS and MDA levels via increased GSH and SOD levels. Finally, KXS protected synapses by increasing the concentrate of ACh through down-regulation of AchE activity and up-regulation of ChAT activity. The mechanisms by which KXS ameliorated depression were as follows (Figure 9). Kaixinsan down-regulated various components of the HPA axis such as CRH, ACTH, and corticosterone. Kaixinsan treatment also resulted in decreased release of TNF-α and IL-6. Furthermore, KXS reduced MDA levels via increased GSH and SOD levels. Moreover, KXS treatment resulted in increased levels of neurotransmitters such as NE, DA, 5-HT, and ACh, which may contribute to protection of synapses. Finally, KXS up-regulated BDNF, which is essential for protection of neurons.

The present study showed that KXS targeted pathways common to AD and depression. Identification of the physiological mechanisms of KXS activity has been limited by limitations of current models of AD and depression. Therefore, more, and diverse, animal models of AD, depression, and BPSD should be used to identify novel targets of KXS.

## Conclusion

This study demonstrated that KXS could significantly protect cognitive function in AD models largely through antioxidant, anti-inflammatory, antiapoptotic, neuroprotective, and synapse protection mechanisms. Furthermore, KXS improved the symptoms of depression in animal models through HPA axis regulation, and antioxidant, anti-inflammatory, synapse protection, and nervous system protection mechanisms. The ability of KXS to effectively treat AD and depression symptoms in animal models suggests that it should be evaluated in clinical studies of AD and BPSD.

## Author Contributions

GZ designed the study, approved the manuscript, and is responsible for this published work. HF, ZX, and XZ collected the data, performed the analyses, and wrote the manuscript.

### Conflict of Interest

The authors declare that the research was conducted in the absence of any commercial or financial relationships that could be construed as a potential conflict of interest.
